# The emerging roles of exosomal long non‐coding RNAs in bladder cancer

**DOI:** 10.1111/jcmm.17152

**Published:** 2022-01-03

**Authors:** Qiang Liu

**Affiliations:** ^1^ Department of Urology Cancer Hospital of China Medical University Liaoning Cancer Hospital and Institute Shenyang Liaoning China

**Keywords:** biomarkers, bladder cancer, exosomes, long non‐coding RNAs, malignant progression

## Abstract

Extracellular vesicles (EVs), especially exosomes, have been reported to play essential roles as extracellular messengers by transporting goods in various diseases, while their potential roles in bladder cancer (BC) still remain to be further studied. BC exhibits a high degree of chemoresistance and metastatic ability, which may be affected by cancer‐derived exosomes that carry proteins, lipids and RNA. To date, the most studied exosomal molecular cargo is long non‐coding RNA (lncRNA). Although there is increasing interest in its role and function, there is relatively little knowledge about it compared with other RNA transcripts. Nevertheless, in the past ten years, we have witnessed increasing interest in the role and function of lncRNA. For example, lncRNAs have been studied as potential biomarkers for the diagnosis of BC. They may play a role as a therapeutic target in precision medicine, but they may also be directly involved in the characteristics of tumour progression, such as metastasis, epithelial‐mesenchymal transition and drug resistance. Cancer cells are on chemotherapy acting. The function of lncRNA in various cancer exosomes has not yet been determined. In this review, we summarize the current studies about the prominent roles of exosomal lncRNAs in genome integrity, BC progression and carcinogenic features.

## 1 INTRODUCTION

Bladder cancer (BC) is one of the most common malignant tumours of the genitourinary system, accounting for the 9th most common malignant tumour in the world.^1^, ^2^ According to pathological classification, 90% of patients with BC have urothelial cancer. About one‐third of these patients are first diagnosed with muscle invasive bladder cancer (MIBC).^3^, ^4^ In some patients, even if the first diagnosis is non‐muscle invasive bladder cancer (NMIBC), 10%–30% of patients progress to MIBC.^4^, ^5^ BC has become a disease that seriously affects human health.^6^, ^7^ At present, its early diagnosis and treatment have made great progress,^8^, ^9^ but its specific mechanism of occurrence and development is still unclear.

In recent years, non‐coding RNAs (ncRNAs) have become a research hotspot. NcRNAs can be divided into housekeeping ncRNAs and regulatory ncRNAs. Among them, regulatory ncRNAs can be mainly divided into microRNA (miRNA), long noncoding RNA (lncRNA) and circular RNA (circRNA).^10^, ^11^, ^12^, ^13^ LncRNA is a general term for single‐stranded nucleotide sequences exceeding 200 bp.^14^ Although it does not have the function of encoding proteins, it can participate in gene regulation at the epigenetic level, transcription level and post‐transcriptional level,^15^, ^16^, ^17^ affect tumour occurrence, development, metastasis and malignant progression of drug resistance.^18^, ^19^, ^20^, ^21^, ^22^, ^23^ Based on the current research on the mechanism of lncRNA, the competitive endogenous ‘ceRNA’ mechanism is the most common type and a widely recognized regulatory mechanism, that is, some ncRNAs have binding sites with microRNAs.^24^, ^25^ The cell acts as a miRNA sponge, thereby releasing the inhibition of miRNA on the target gene, thereby increasing the expression level of the target gene.^26^, ^27^, ^28^, ^29^ For instances, elevated LINC00909 can promote tumour progression of ovarian cancer via regulating the miR‐23b‐3p/MRC2 axis.^30^ Long noncoding RNA IL6‐AS1 is upregulated in chronic obstructive pulmonary disease and is interrelated to interleukin 6 via sponging miR‐149‐5p and regulating early B‐cell factor 1 expression^31^; Long non‐coding RNA (lncRNA) DLEU2 can drive epithelial‐mesenchymal transition (EMT) genes and glycolysis in endometrial cancer through modulating the miR‐455/HK2 and EZH2/miR‐181a pathway.^32^

Exosomes are small extracellular vesicles (EVs) (30–100 nm in diameter).^33^, ^34^ They are secretory vesicles containing cytoplasmic protein and RNA in a bilayer of phospholipids and exist in all eukaryotic and prokaryotic cells.^35^, ^36^, ^37^ Through their role as transporters, exosomes form complex networks that connect tumour cells in the tumour microenvironment and play a crucial role in these networks.^38^, ^39^, ^40^ Carrying substances through autocrine, paracrine, endocrine and other signalling pathways, exosomes transport specific proteins, DNA and RNA to recipient cells, thereby regulating the biological characteristics of the recipient cells.^41^, ^42^, ^43^, ^44^ For example, exosomes can promote tumour development, inhibit tumour cell apoptosis and immune escape, stimulate tumour angiogenesis and transfer genetic material.^45^, ^46^, ^47^, ^48^, ^49^, ^50^ Recently, it has been shown that the release of exosomes may act as a vital role in the chemotherapy resistance of cancer cells by mediating the transfer of drugs, nucleic acids and proteins.^51^, ^52^, ^53^

In recent years, studies have shown that exosomal‐lncRNAs also play an indispensable role in the occurrence and progression of many cellular processes. Besides, exosomal lncRNAs can regulate the tumour microenvironment by modulating the expression of various key signalling pathways and molecular and play important regulatory roles in cancer metastasis. Moreover, due to their specificity and sensitivity, exosomal lncRNAs can also be released into tumour microenvironments and act as potential tumour markers. Ni et al. show that breast cancer‐derived exosomal lnc‐SNHG16 can enhance the activation of the TGF‐β1/SMAD5 pathway through the miR‐16‐5p/SMAD5 regulatory axis, thereby inducing the expression of CD73 in Vδ1 T cells and leading to malignant tumour progression.^54^ Li et al. identify that the expression of Lnc‐FMR1‐AS1 is increased in the tissues of patients with oesophageal cancer and is related to the poor prognosis of patients.^55^ Lnc‐FMR1‐AS1 can be packaged into exosomes and released into the tumour microenvironment, and maintain the dynamic interconversion state of tumour stem cells by activating TLR7‐NFκB signalling and up‐regulating c‐Myc levels in recipient cells. Guo et al. found that we can detect the occurrence of gastric cancer (GC) and predict the later progression of GC by detecting the expression of circulating exosomes lncRNA‐GC1.^56^ Combining the detection of circulating exosomes lncRNA‐GC1 with endoscopy can improve the early diagnosis rate of GC. Lin et al. reveal that the AUC values of lncUEGC1 in distinguishing EGC patients from healthy individuals and patients with precancerous chronic atrophic gastritis were 0.8760 and 0.8406, respectively, which were higher than the diagnostic accuracy of carcinoembryonic antigen and were a good marker for early diagnosis of GC.^57^

In this review, we focused on the latest evidence of major exosomal lncRNAs related to BC, and discussed the latent biological role of exosomal lncRNAs in the development, treatment and clinical applications of BC.

## 2 BIOGENESIS AND CHARACTERISTICS OF EXOSOME

### 2.1 Biochemical characteristics of exosomes

**FIGURE 1:**
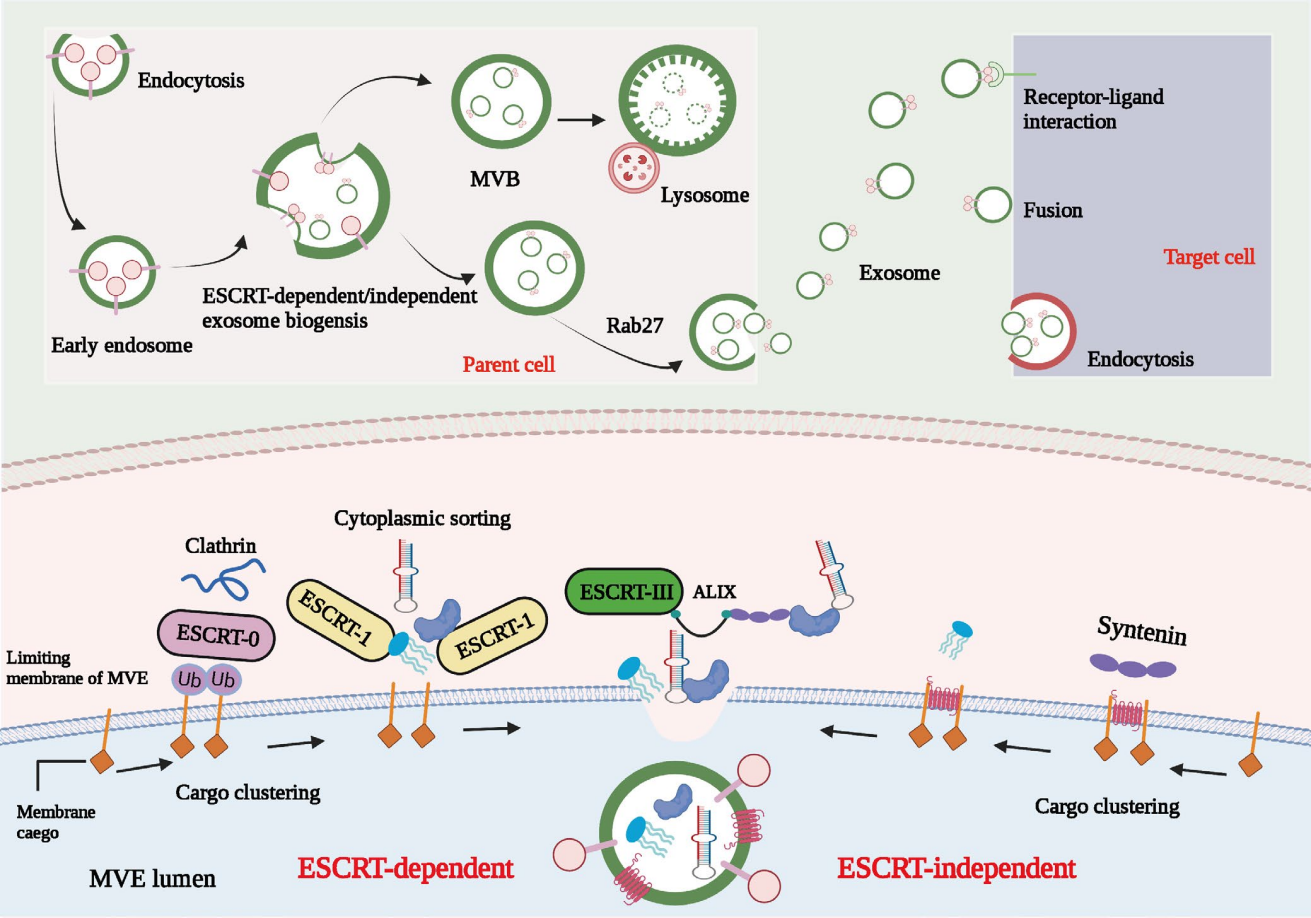
The biological process of exosome biogenesis and release. The biogenesis of exosomes begins with endosomes formed by plasma membrane endocytosis, and then early endosomes mature into multivesicular bodies (MVB). Exosomes are formed as intraluminal vesicles (ILV) in MVB through endosome‐sorting complexes required for transport (ESCRT)‐dependent or ‐independent pathways. MVB can be degraded by fusing with lysosomes or fused with the plasma membrane, leading to the secretion of ILV (exosomes). Target cells can take up exosomes through endocytosis, fusion with plasma membrane or ligand/receptor interaction. Cells can package different substances (including lncRNAs) into exosomes through ESCRT‐dependent manner and ESCRT‐independent manner

There are two main secretion mechanisms of exosomes: continuous secretion dependent on Golgi and induced secretion.^34^ Different subtypes of exosomes may have different release mechanisms and carry different cargo components. A large number of proteins are enriched on and in the exosomal membrane, such as membrane transport and membrane fusion proteins (such as GTPases, Annexins and Flotillin), proteins required for the synthesis of multivesicles (such as tumour susceptibility gene 101), four Transmembrane proteins (such as CD9, CD63, CDS1), apoptosis‐linked gene 2 interacting protein X (ALIX), heat shock proteins (such as HSP70, HSP90).^45^, ^58^, ^59^, ^60^ Exosomes carry many nucleic acid molecules, such as miRNA, ncRNA and mRNA.^47^, ^50^, ^58^ In addition, it also carries cytokines and growth factor proteins similar to the source cells.^60^, ^61^ The biological process of exosome biogenesis and release was showed in Figure 1.

The separation methods of exosomes have not yet been unified, including sucrose gradient centrifugation, differential ultracentrifugation, filtration centrifugation, immunoaffinity capture technology, chromatography technology, microfluidic chip technology and PEG polymer precipitation.^62^, ^63^, ^64^ The appropriate combination of these technologies may be effective. Even better, there are more commercial kits based on the above principles. The gold standard method is differential ultracentrifugation.^65^, ^66^ The exosomes obtained by the sucrose gradient centrifugation method have high purity, but the preliminary preparation is time‐consuming, the extraction process is very time‐consuming and the yield is low. Exosomes can be stored at 4°C for a short term (within 1–2 days) and stored at 80°C for a long term.

The identification of exosomes relies on morphological observation and protein composition analysis.^53^, ^67^, ^68^ Observe the morphology of exosomes under an electron microscope. It can be seen that they are cup‐shaped or flat balloons. Nanoparticle tracking analysis (NTA) can also be used to measure their diameters; protein composition analysis usually uses Western blot to detect exosomes. The protein expression level of body enrichment, as usual, chooses to detect CD63 and CD81.

### 2.2 Exosomes and tumours

Exosomes were first discovered to participate in antigen presentation and immune activation and suppression.^69^, ^70^ Mast cells transport their mRNA and miRNA to recipient cells through the released exosomes, and translate proteins in the recipient cells, thus proving that exosomes have the function of transporting substances.^71^, ^72^ The lipid bilayer membrane of exosomes reduces the degradation of exosomes by proteases and ribonuclease, and is shed from the cell membrane through autocrine, paracrine and endocrine secretion pathways.^73^, ^74^, ^75^ The membrane carries proteins and nucleic acid signal molecules. Body–ligand interaction, direct membrane fusion and endocytosis (or phagocytosis) are 3 ways to transfer signals from exosomes to recipient cells, and participate in intercellular communication, angiogenesis, immune response and tumour growth physiology and pathology process.^76^

Exosomes participate in the composition of the tumor microenvironment and promote soluble proteins, nucleic acids, functional transmembrane proteins, chemokine receptors, epidermal growth factor receptors to mediate tumorigenesis, growth, tumor vascular growth, tumor metastasis, tumor immune escape, formation of tumor microenvironment.^77^, ^78^ Tumour cells release exosomes, and the signal molecular characteristics they carry can reflect the phenotype of tumour cells, such as tumour‐specific antigen proteins and RNA. They have great potential as tumour diagnostic markers.^79^, ^80^ At the same time, tumour cells can excrete anti‐tumour drugs by secreting exosomes, resulting in multiple tumours. Exosomes are closely related to tumours, participating in tumour formation, metastasis, drug resistance, evading immune surveillance and can also assist in diagnosis and treatment.^81^, ^82^

## 3 EXOSOMAL‐LNCRNA IN BLADDER CANCER

### 3.1 The potential biological role of exosomal lncRNAs in BC

**FIGURE 2:**
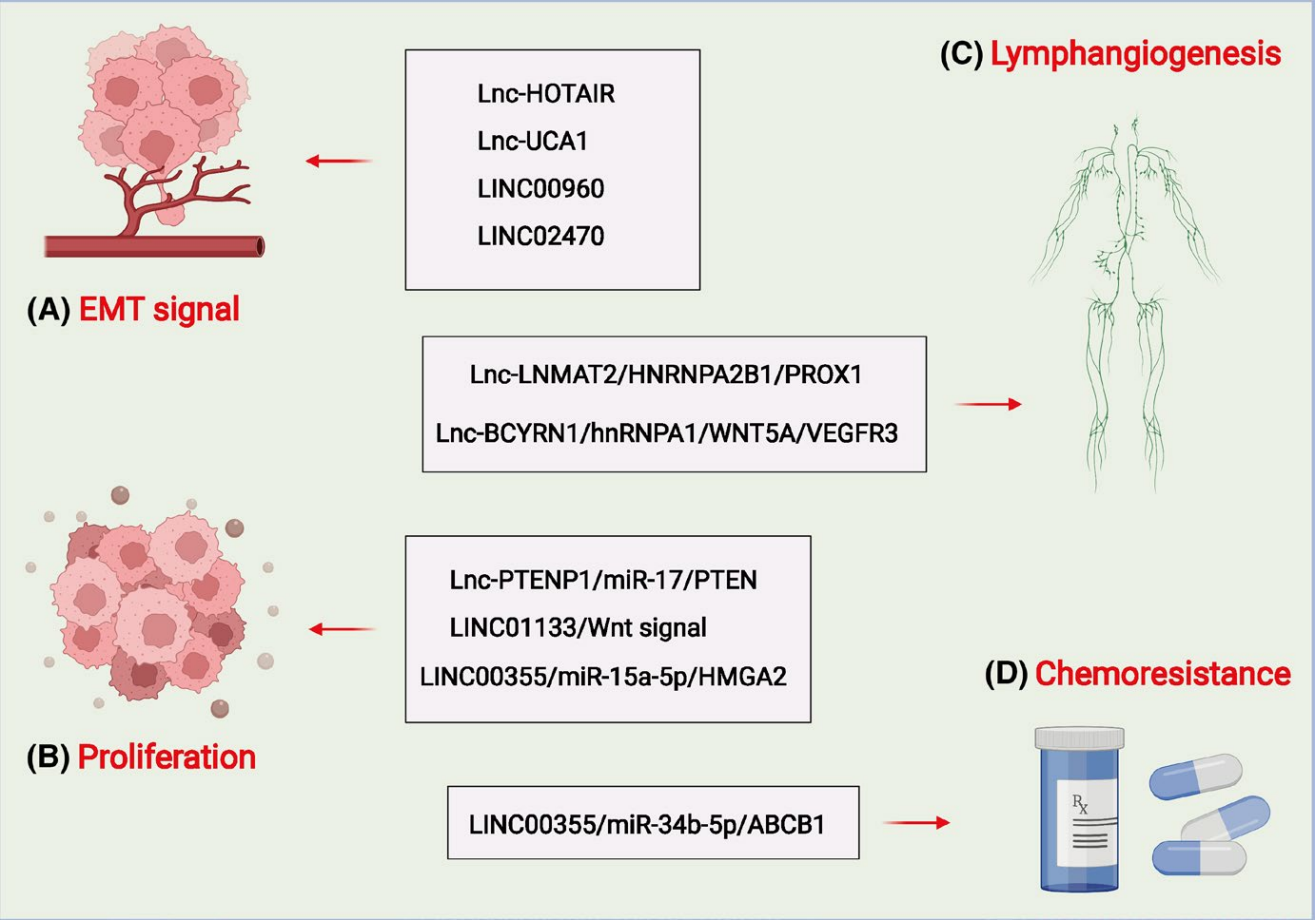
Role and functions of deregulated exosomal lncRNAs in BC progression. Exosomal lncRNAs can participate in the progression of BC by regulating the EMT (A), cell proliferation (B), lymphangiogenesis (C) and chemoresistance (D). Long noncoding RNA, lncRNA; bladder cancer, BC; HOX transcript antisense RNA, HOTAIR; epithelial‐to‐mesenchyme transition, EMT; urothelial cancer‐associated 1, UCA1; phosphatase and tensin homologue deleted on chromosome ten, PTEN; micro RNA, miRNA; lymph node metastasis‐associated transcript 2, LNMAT2; heterogeneous nuclear ribonucleoprotein A2B1, hnRNPA2B1; prospero homeobox 1, PROX1; ATP binding cassette subfamily B member 1, ABCB1; brain cytoplasmic RNA 1, BCYRN1; integration site family member 5A, Wnt5a; Vascular endothelial growth factor receptor 3, VEGF3; cancer‐associated fibroblasts, CAFs; human lymphatic endothelial cells, HLECs. Long noncoding RNA, lncRNA; bladder cancer, BC; orthodenticle homeobox 2 antisense 1, OTX2‐AS1; metastasis‐associated lung adenocarcinoma transcript 1, MALAT1; prostate cancer‐associated transcript 1, PCAT1; SPRY4 intronic transcript 1, SPRY4‐IT1; phosphatase and tensin homolog pseudogene 1, PTENP1; small nucleolar RNA host gene 16, SNHG16; H19 imprinted maternally expressed transcript, H19

Previous studies have shown that not only exosomal miRNAs serve as a vital role in the occurrence and progression of tumours,^83^, ^84^, ^85^, ^86^ but also lncRNAs in exosomes have important biological effects. We summarized the biological role of exosomal‐lncRNAs in BC (Figure 2; Table 1).

**TABLE 1:** Potential role and mechanism of exosomal lncRNA in BC

LncRNAs	Parent cell/source	Target cell	Mechanism	Biological function	Reference
HOTAIR	Urine	T24 and TCC‐SUP	Regulate EMT signalling pathway	Promote cell migration and invasion	[91]
UCA1	5637	UMUC2	Regulate EMT signalling pathway	Promote tumor growth and progression	[92]
PTENP1	293A	J82 and EJ	PTENP1/miR‐17/PTEN	Increase cell apoptosis and reduce the ability to invade and migrate	[97]
LNMAT2	UMUC3/5637	HLEC	LNMAT2/HNRNPA2B1/PROX1	Stimulated HLEC tube formation and migration enhanced tumour lymphangiogenesis	[102]
LINC00960 LINC02470	T24 and J82	TSGH‐8301	Regulate EMT	Enhance the cell viability, migration, invasion and clonogenicity	[93]
LINC00355	CAFs	T24 and 5637	LINC00355/miR‐34b‐5p/ABCB1	promotes BC cell resistance to cisplatin	[107]
LINC01133	SV‐HUC‐1	T24 and J82	Regulate Wnt signalling pathway	Restrain cell viability, proliferation, migration,	[98]
BCYRN1	UMUC3/5637	HLEC	BCYRN1/hnRNPA1/WNT5A/VEGFR3	Promote tube formation and migration of HLECs, lymphangiogenesis and LN metastasis of BCa‐HOTAIR	[103]
LINC00355	CAFs	T24 and 5637	LINC00355/miR‐15a‐5p/HMGA2	Promote BC cell proliferation and invasion	[107]

Abbreviations: ABCB1, ATP‐binding cassette subfamily B member 1; BC, bladder cancer; BCYRN1, brain cytoplasmic RNA 1; CAFs, cancer‐associated fibroblasts; EMT, epithelial–mesenchyme transition; HLECs, human lymphatic endothelial cells; hnRNPA2B1, heterogeneous nuclear ribonucleoprotein A2B1; HOTAIR, HOX transcript antisense RNA; lncRNA, Long noncoding RNA; LNMAT2, lymph node metastasis‐associated transcript 2; miRNA, micro RNA; PROX1, prospero homeobox 1; PTEN, phosphatase and tensin homologue deleted on chromosome ten; UCA1, urothelial cancer‐associated 1; VEGF3, Vascular endothelial growth factor receptor 3; Wnt5a, integration site family member 5A.

#### 3.1.1 Exosomal lncRNAs and epithelial‐mesenchymal transition (EMT) in BC

Epithelial‐mesenchymal transition is intimately interrelated to the development of tissues or organs during embryogenesis. Additionally, this phenomenon is significantly associated with tumour development^87^ and is a trigger for invasion, migration and acquisition of stem cell‐like phenotype in cells of diverse cancers, including BC.^88^ EMT can promote the gain of epithelial stem cell properties, association with stem‐like cell markers and generation of cancer stem cells.^89^ EMT is established by EMT‐inducible transcription factors, such as ZEB1, ZEB2, Snail, Slug and Twist. These transcription factors can inhibit the expression of epithelial marker E‐cadherin and increase the mesenchymal marker N‐cadherin expression to promote EMT.^90^ Besides, EMT can also be regulated by several diverse upstream regulators, including signalling molecules and exosomal lncRNAs by various mechanisms.

Studies have shown that exosomal lncRNAs can regulate the EMT of BC (Figure 2A). Berondo et al. showed that HOX transcribed antisense RNA (HOTAIR) and several tumour‐related lncRNAs were rich in biological fluids, such as urine from EU patients with urothelial bladder cancer (UBC) with highly aggressive muscle diseases (HGMI pT2‐pT4) (EU).^91^ Inhibition of HOTAIR in UBC cell lines could reduce cell migration and invasion. Besides, the loss of HOTAIR expression in UBC cell lines altered the expression of EMT‐related genes. They also utilized RNA sequencing to identify four 4 EU‐rich ncRNAs from UBC patients. Xue et al. proved that hypoxic BC cells could reshape the tumour microenvironment to promote tumour growth and progression, and secrete carcinogenic exosomes rich in lncRNA‐UCA1. Besides, exosomal lnc‐UCA1 might be used as a latent diagnostic biomarker for BC.^92^ Huang and colleagues reported that LINC00960 and LINC02470 from high‐grade BC cell exosomes could promote the malignant behaviour of receptor low‐grade BC cells and induce EMT by up‐regulating receptor β‐catenin, Notch and Smad2/3 signalling.^93^

#### 3.1.2 Exosomal lncRNAs and cell proliferation in BC

Cell proliferation is a precision control process, which is vital for embryonic and postnatal development.^94^ Under pathological conditions, abnormal cell proliferation is a central mechanism attributing to disease progressions. Abnormal cell proliferation includes both abnormal cell division and abnormal cell differentiation.^95^ Besides, cell proliferation is also a main characteristic of cancer cells and the base of metastasis.^96^

Studies have shown that exosomal lncRNAs can regulate the cell proliferation of BC (Figure 2B). Zheng and colleagues showed that exosomal PTENP1 was a novel biomarker that could be applied for clinical detection of BC.^97^ Exosomes secreted by normal cells could transfer PTENP1 to BC cells and suppress cell growth and metastasis. The results indicated that exosomal PTENP1 might participate in the communication between normal cells and BC cells in the process of BC carcinogenesis. In addition, exosome‐mediated could transfer LINC01133 inhibits the progression of BC by regulating the Wnt signalling pathway.^98^

#### 3.1.3 Exosomal lncRNAs and lymphangiogenesis in BC

Tumour‐induced lymphangiogenesis plays an essential role in promoting tumour growth and metastasis.^99^ Tumour‐associated lymphatic vessel density is closely correlated with sentinel lymph node metastasis, distant metastasis and patient survival.^100^ In addition, lymph endothelial cells can interact with various immune cells to modulate immune cell activity.^101^ By the above‐mentioned means, lymphatic vessels can act as vital roles in the malignant progression of tumours.

Studies have shown that exosomal lncRNAs can regulate the lymphangiogenesis of BC (Figure 2C). Chen et al. identified that lncRNA lymph node metastasis‐associated transcript 2 (LNMAT2) could stimulate the tube formation and migration of human lymphatic endothelial cell (HLEC) and strengthen lymphatic vessels of tumour generation and LN metastasis.^102^ Mechanically, exosomal LNMAT2 could be secreted by BC cells and interact with the heterogeneous ribonucleoprotein A2B1 (hnRNPA2B1). Subsequently, the expression of prospero homeobox 1 (PROX1) was up‐regulated by the recruitment of hnRNPA2B1, leading to lymphangiogenesis and lymphatic metastasis. Zheng et al. discovered through which exosomal BCYRN1 synergistically enhances lymphatic metastasis induced by VEGF‐C/VEGFR3 signalling from BCa, indicating that BCYRN1 may serve as an encouraging therapeutic target for BCa patients.^103^

#### 3.1.4 Exosomal lncRNAs and chemoresistance in BC

Chemoresistance can be divided into primary drug resistance and multiple drug resistance (MDR). The former refers to cancer cells that are resistant to induced drugs, whereas the latter refers to cancer cells that develop resistance to induced drugs or other chemotherapeutic agents.^104^ The establishment of chemoresistance in cancer cells involves various mechanisms, including downregulation of apoptosis, increased DNA repair, altered drug targets and overexpression of MDR proteins.^105^, ^106^

Studies have shown that exosomal lncRNAs can regulate the chemoresistance of BC **(** **Figure** **2D** **)**. Luo et al. proved that the CAF‐derived exosome LINC00355 could promote the resistance of BC cells to cisplatin by regulating the miR‐34b‐5p/ABCB1 axis.^107^

### 3.2 Exosomal lncRNAs could act as diagnostic and prognostic Biomarkers in BC

Previous studies have shown that exosomal lncRNAs play a crucial role in the early diagnosis and prognostic evaluation of tumours.^108^, ^109^, ^110^, ^111^ We summarized the diagnostic and prognostic value of exosomal‐lncRNAs in BC (Table 2).

**TABLE 2:** Potential of exosomal lncRNA as diagnostic and prognostic tool in BC

LncRNA	Source of exosome	Exosome isolation techniques	Biomarker potential	References
HYMA1, LINC00477, LOC100506688 and OTX2‐AS1	Urine	Ultracentrifugation	Biomarkers for BC prognosis	[91]
UCA1	Serum	ExoQuick solution	A biomarker for BC diagnosis	[92]
MALAT1, PCAT1 and SPRY4‐IT1	Urine	Ultracentrifugation	Biomarkers for BC diagnosis and prognosis	[112]
PTENP1	Plasma	ExoQuick solution	A biomarker for BC diagnosis	[97]
PCAT‐1, UBC1 and SNHG16	Serum	ExoQuick solution	Biomarkers for BC diagnosis and prognosis	[113]
H19	Serum	ExoQuick solution	A biomarker for BC diagnosis and prognosis	[114]
UCA1‐201, HOTAIR, HYMA1 and MALAT1	Urine	Ultracentrifugation	Biomarkers for BC diagnosis	[115]

Abbreviations: BC, bladder cancer; H19, H19 imprinted maternally expressed transcript; HOTAIR, HOX transcript antisense RNA; lncRNA, Long noncoding RNA; MALAT1, metastasis associated lung adenocarcinoma transcript 1; OTX2‐AS1, orthodenticle homeobox 2 antisense 1; PCAT1, prostate cancer associated transcript 1; PTENP1, phosphatase and tensin homolog pseudogene 1; SNHG16, small nucleolar RNA host gene 16; SPRY4‐IT1, SPRY4 intronic transcript 1; UCA1, urothelial cancer associated 1.

Berondo et al. detected the expression level of lncRNAs in the original 8 patients plus two additional patient UEs (n = 10 UBC patient UEs) compared to the original 3 HV UEs and additional 4 HV UEs (n = 7 HVs) ‐seq data by qRT‐PCR.^91^ The results showed that UE of patients with HGMI disease (pT2‐pT4) is rich in lncRNA HYMA1, LINC00477, LOC100506688 and OTX2‐AS1. It identified that UEs from UBC patients contain ncRNA and might eventually play a role in the prognosis of BC. Xue et al. utilized the ROC curve to analyse the diagnostic value of exosomal UCA1 in BC patients’ serum. The results showed that exosomal UCA1 could be a promising potential biomarker for BC.^92^ Moreover, three differently expressed lncRNAs (MALAT1, PCAT‐1 and SPRY4‐IT1) were established to diagnose BC, and they are identified to be potential biomarkers.^112^ In addition, Kaplan–Meier analysis showed that the up‐regulation of PCAT‐1 and MALAT1 was interrelated to poor recurrence‐free survival (RFS) of NMIBCs, and more the variable Cox proportional hazard regression analysis showed that the overexpression of PCAT‐1 in exosomes was an independent prognostic factor of NMIBC RFS. Zheng and colleagues found that exosomal PTENP1 could distinguish BC patients from healthy controls.^97^ In addition, a set of three lncRNAs (PCAT‐1, UBC1 and SNHG16) were finally identified by a multiple logistic regression model to provide BC with high diagnostic accuracy.^113^ Additionally, Kaplan–Meier analysis revealed that NMIBC patients with high UBC1 expression had a significantly lower recurrence‐free survival rate. Cox multivariate analysis showed that UBC1 was independently correlated to tumour recurrence in NMIBC. Wang et al. showed that the detection of exosomal H19 serum clarifies the use of exosomal lncRNA as a non‐invasive diagnostic and prognostic biomarker for BC patients.^114^ Yu et al. selected four lncRNAs, namely UCA1‐201, HOTAIR, HYMA1 and MALAT1, to form a set of urine biomarkers of BC.^115^ With the help of this panel, BC patients could be distinguished from patients with allantoicitis, with sensitivity and specificity reaching 95.7% and 94.3%, respectively. Finally, they confirmed the applicability of the four lncRNA combinations in an independent validation study involving 60 patients with BC and 60 patients with allantoicitis.

## 4 FUTURE EXPECTATIONS OF EXOSOME IN BC

No matter how hard the current multidisciplinary treatment is, the high recurrence rate of BC is still the biggest obstacle for treatment.^116^, ^117^, ^118^ The important role of exosomes‐mediated signal transduction in cancer progression makes exosomes a potential new therapeutic target, which focuses on inhibiting the key components of the tumour cell communication network. Exosomes are expected to play an important role in the treatment of BC patients, help early diagnosis and monitoring and provide accurate predictive markers.

### 4.1 Exosomes are used to develop carriers for the transportation of anti‐cancer drugs

The lipid bilayer membrane of exosomes can protect nucleic acids and proteins in the membrane from being degraded. At the same time, there are recognition molecules on the membrane, and exosomes can become a good carrier for targeted drug delivery. It can accurately transport interfering RNA, suicide mRNA, protein, miRNA and drugs. Despite the huge therapeutic potential of exosomes, the field still needs new in vivo models and powerful imaging systems to track the pathways of the synthesis, release, transportation and function of single‐cell exosomes.

### 4.2 Inhibition of tumour progression and metastasis by targeting tumour‐derived exosomes

Exosomes participate in the formation of the tumour microenvironment, and the signal transduction between tumour cells can inhibit the occurrence and development of tumours. There are currently several potential strategies. By interfering with the pathway components involved in the formation of exosomes (such as ESCRT, neural Amide) or release (such as Rab27, ARF6, RhoA) to inhibit the biogenesis or release of exosomes. Remove exosomes from the circulation through extracorporeal hemofiltration. Block those exosomes involved in exosome binding or internalization. Exosomal ligands (such as four transmembrane proteins) or cell surface receptors (such as HSPG) inhibit the uptake of exosomes by recipient cells.

### 4.3 Future prospects of exosomal lncRNAs in BC

At present, studies have found that exosomal lncRNA has an important biological role in BC, but more research is still needed to explore the clinical translational value of exosomal lncRNAs in BC. Many studies have confirmed that exosomal lncRNAs can promote the malignant progression of tumours by promoting angiogenesis.^119^, ^120^, ^121^ In addition, exosomal lncRNA can also mediate immunosuppressive microenvironments,^122^, ^123^ regulate cell radioresistance^124^ and mediate metabolic reprogramming,^125^ but there is no relevant research report in BC. In the future, more research should be done to explore the role of exosomal lncRNAs in the above aspects and the prospects of clinical application. The role of Exosomal lncRNAs in tumour liquid biopsy has been confirmed by research. The current research on exosomal lncRNAs in the diagnosis of BC is mostly focused on the experience of a single centre, and the diagnostic potential of exosomal lncRNAs can only be tested by further verification in a multi‐centre joint study.

## 5 CONCLUSION

Due to its very aggressive nature, BC has the lower survival rate of urology cancers.^126^, ^127^, ^128^ This extremely high mortality rate is primarily the result of its early asymptomatic development, so it is diagnosed as late.^129^, ^130^ Therefore, there is an urgent need for new diagnostic tools and new treatment strategies. In recent years, the potential role of lncRNAs as biomarkers, therapeutic targets and therapeutics in cancer research have attracted increasing interest. However, the pathophysiological function of lncRNAs still remains unknown, and whether they are the cause or consequence of cancer remains to be determined. Additionally, the same lncRNA can play completely distinct roles in various cancer environments, which makes the characterization of lncRNA particularly difficult. Although most studies are still in the preclinical stage, the diagnostic and prognostic applications of lncRNA related to exosomes are very promising for BC treatment. New advances in lncRNAs‐related studies in specific fields, such as bioinformatics, pharmacokinetics, and improved nanotechnology to deliver lncRNAs‐containing exosomes to the tumour microenvironment, will lay the foundation for future clinical applications. Understanding the function and role of lncRNAs is essential for their effective use as biomarkers, precision medicine or therapeutic targets.

## CONFLICT OF INTEREST

The authors declare that there are no conflicts of interest.

## AUTHOR CONTRIBUTIONS

**Qiang Liu:** Original draft preparation, allocation, revision, supplement and edition.

## Data Availability

The data in the current study are available from the corresponding authors on reasonable request.
